# The Effects of Dabigatran and Rivaroxaban on Markers of Polymorphonuclear Leukocyte Activation

**DOI:** 10.3390/ph11020046

**Published:** 2018-05-14

**Authors:** Guy A. Richards, Annette Theron, Gregory Tintinger, Ronald Anderson

**Affiliations:** 1 Department of Critical Care, Faculty of Health Sciences, University of the Witwatersrand, Johannesburg 2193, South Africa; 2 Department of Immunology, Faculty of Health Sciences, University of Pretoria, Pretoria 0001, South Africa; atheron@up.ac.za (A.T.); ronald.anderson@up.ac.za (R.A.); 3 Tshwane Academic Division of the National Health Laboratory Service of South Africa, Pretoria 0001, South Africa; 4 Department of Internal Medicine, Faculty of Health Sciences, University of Pretoria, Pretoria 0001, South Africa; greg.tintinger@up.ac.za

**Keywords:** dabigatran, rivaroxaban, inflammation, ischemic heart disease, polymorphonuclear leukocytes

## Abstract

Dabigatran is an oral direct thrombin inhibitor, and rivaroxaban, a factor Xa inhibitor. Dabigatran has been implicated in the etiology of acute coronary syndromes and as these occur following inflammatory changes in the endothelium, we investigated the inflammatory potential of these agents in vitro. In order to do so, polymorphonuclear leukocytes (PMNL) were isolated from heparinized venous blood from non-smoking, healthy adults and exposed to dabigatran or rivaroxaban (0.5–10 µM). Generation of reactive oxygen species (ROS), elastase release, cytosolic Ca^2+^ fluxes, neutrophil extracellular trap (NET) formation and cell viability were measured using chemiluminescence, spectrophotometric and flow cytometric procedures respectively. However, with the exception of modest inhibitory effects on elastase release, neither agent at concentrations of up to 10 µM affected these markers of PMNL activation. Although no pro-inflammatory effects of dabigatran nor any difference between the two test agents were detected in vitro, the existence of a pro-inflammatory mechanism involving the generation of thrombin during dabigatran therapy cannot be fully excluded.

## 1. Introduction

Atherosclerosis is an inflammatory condition in which endothelial injury predisposes to endothelial lipid accumulation and plaque formation [1]. Risk factors for atherosclerosis include hypertension, diabetes, cigarette smoking, hyperlipidemia and possibly exposure to atmospheric pollutants. However, individuals without apparent risk factors also develop vascular disease, including coronary heart disease (CHD), which in both settings is accompanied by low-grade systemic inflammation associated with an increase in cytokines and other inflammatory biomarkers. Accordingly, inflammation, including platelet-driven polymorphonuclear leukocyte (PMNL) inflammation, plays a critical role in the development and rupture of atherosclerotic plaques [2,3,4,5,6].

In this context, the novel oral anticoagulants (NOACs) represent a breakthrough in the management of thrombotic conditions. The mechanisms of therapeutic action of these agents involve direct inhibition of thrombin (dabigatran) or inhibition of factor Xa (rivaroxaban, apixaban, edoxaban) [7]. Although NOACs have been used successfully in diverse conditions, including venous thromboembolism (both deep vein thrombosis and pulmonary embolism) and stroke prevention in atrial fibrillation, there have been concerns that direct thrombin inhibitors may predispose to, or cause, coronary thrombosis [8]. These concerns originated with the use of the intravenous thrombin inhibitors lepirudin, argatroban, desidurin and bivalirudin with several studies suggesting that bivalirudin is associated with coronary thromboses (number needed to harm (NNH) between 50 and 111) [8]. This paradoxical effect is particularly evident with the new oral anticoagulant, dabigatran. Although an effective anticoagulant, a number of studies have suggested an association between dabigatran and myocardial thrombosis as reviewed by Davidson [8]. This contention is apparently supported by a study of 18,000 patients with atrial fibrillation, which compared dabigatran to warfarin [9]. The authors reported an increased risk of myocardial infarction (MI) with dabigatran of 1.38 and 1.35 times that of warfarin at the two dosing schedules of 150 mg and 110 mg bid (*p* = 0.05 and 0.07, NNH 476 and 526, respectively) [9]. However, the statistical significance that was achieved with the higher dose was subsequently corrected by the authors who adjusted the numbers of patients with an MI in each group [10]. Similarly, in two clinical trials assessing the efficacy of dabigatran in venous thromboembolism (VTE), the pooled odds ratio (OR) for acute coronary syndromes was 1.8 (95% CI, 0.6–6.2; NNH 313), which, although increased, was non-significant [8,11,12]. Moreover, Boehringer-Ingelheim GmbH, the manufacturers of dabigatran, concluded that the incidence of MI with warfarin (when used to prevent stroke in patients with atrial fibrillation or for acute VTE treatment or prevention) is significantly lower than that observed with the higher dose of dabigatran (OR of 1.4 and 95% CI, 1.1–1.9).

There have also been other studies to which smaller numbers of patients were recruited, which reported increased frequencies of MIs in patients treated with dabigatran compared to warfarin, while a larger study evaluating the use of dabigatran in patients with valve replacements was stopped prematurely due to an increased number of adverse events, including stroke in the study group [8]. In addition, a recent meta-analysis based on the pooled results of seven randomized, controlled trials, comparing dabigatran with many other anticoagulants, reported that dabigatran may increase the relative risk of MI by 27–33% [13].

Although the apparent paradoxical effects of dabigatran on thrombin generation may contribute to platelet-driven PMNL activation, the direct effects of this agent on the reactivity of these cells remain largely unexplored [14,15]. Accordingly, in this study we have assessed the effects of dabigatran, together with rivaroxaban as a comparator, on the production of reactive oxygen species (ROS), elastase release, cytosolic Ca^2+^ fluxes, and extracellular trap formation following activation of human PMNL in vitro.

## 2. Materials and Methods

### 2.1. Chemicals and Reagents

The active forms of dabigatran (C_25_H_25_N_7_O_3_; 3-[[2-[(4-carbamimidoylanilino)methyl]-1-methylbenzimidazole-5-carbonyl]-pyridin-2-ylaminopropanoic acid) and rivaroxaban (C_19_H_18_ClN_3_O_5_S; 5-chloro-*N*-[[(5S)-2-oxo-3-[4-(3-oxomorpholin-4-yl]-1,3-oxazolidin-5-yl]methyl]thiophene-2-carboxamide) were provided by Boehringer Ingelheim GmbH (Ingelheim, Germany) and Bayer Pharma AG (Leverkusen, Germany) respectively and each dissolved in dimethylsulfoxide (DMSO) to give stock concentrations of 10 mM. In the assays described below, both agents were used at final concentrations of 0.5–10 μM together with the appropriate solvent control systems (0.1% DMSO, final). Unless indicated, all other chemicals and reagents were purchased from Sigma-Aldrich, St. Louis, MO, USA.

Polymorphonuclear leukocytes (PMNL) were activated with the phorbol ester, phorbol 12-myristate 13-acetate (PMA), in assays of generation of ROS and neutrophil extracellular trap (NET) formation, or with the chemoattractant, *N*-formyl-l-methionyl-l-leucyl-l-phenylalanine (fMLP) in combination with cytochalasin B (CB) in assays cytosolic Ca^2+^ fluxes and degranulation respectively.

### 2.2. Preparation of PMNL

The study was approved by the Faculty of Health Sciences Research Ethics Committee of the University of Pretoria, Pretoria, South Africa, and prior informed consent was obtained from all blood donors. PMNL were isolated from heparinized venous blood (5 units of preservative-free heparin per mL of blood) from non-smoking, healthy, adult volunteers. Subjects completed a detailed health questionnaire and underwent a medical examination by an experienced qualified nursing sister prior to venepuncture.

PMNL were separated from mononuclear leukocytes by centrifugation on Histopaque-1077 (Sigma Diagnostics, St. Louis, MO, USA) cushions at 400× *g* for 25 min at room temperature. The resultant pellets were suspended in phosphate-buffered saline (PBS, 0.15 M, pH 7.4) and sedimented with 3% gelatin to remove most of the erythrocytes. Following centrifugation (280× *g* at 10 °C for 10 min), residual erythrocytes were removed by selective lysis with 0.83% ammonium chloride at 4 °C for 10 min. The PMNL, which were routinely of high purity (>90%) and viability (>95%), were re-suspended to 1 × 10^7^ cells/mL in PBS and held on ice until used.

### 2.3. Measurement of Reactive Oxygen Species (ROS)

These were measured using lucigenin (bis-*N*-methyl-acridinium nitrate) and luminol (5-amino-2,3-dihydro-1,4-phthalazine dione)-enhanced chemiluminescence (CL) procedures that predominantly detect superoxide and ROS generated by the myeloperoxidase/H_2_O_2_/halide system respectively [16].

Briefly, PMNL (10^6^ cells) were pre-incubated for 15 min at 37 °C, without and with dabigatran or rivaroxaban (0.5–10 μM) in 900 μL of indicator-free Hanks’ balanced salt solution (HBSS, pH 7.4) containing either lucigenin (0.2 mM) or luminol (0.1 mM), followed by addition of either 100 μL of HBSS (unstimulated control systems) or PMA [25 nanograms (ng)/mL)] and CL responses recorded using an LKB Wallac 1251 Luminometer (Turku, Finland). The results, which are expressed in millivolts/second (mV/s), are the peak values for PMA-activated systems that were reached at around 10 min after addition of the stimulant.

### 2.4. Elastase Release

PMNL degranulation was measured according to the extent of release of the primary granule enzyme, elastase. PMNL were pre-incubated at a concentration of 2 × 10^6^/mL in HBSS with and without dabigatran or rivaroxaban (0.5–10 μM) for 10 min at 37 °C. The cells were then activated with fMLP (1 μM) in combination with CB (0.5 μM) and incubated for 10 min at 37 °C. The tubes were then transferred to an ice bath, followed by centrifugation at 400× *g* for 5 min to pellet the cells. The PMNL-free supernatants were then decanted and assayed for elastase using a micro-modification of a standard colorimetric procedure [17]. Briefly, 125 μL of supernatant was added to the elastase substrate, *N*-succinyl-l-alanyl-l-alanyl-l-alanine-*p*-nitroanilide (3 mM) in 0.05 M Tris-HCl (pH 8.0), and elastase activity monitored spectrophotometrically at a wavelength of 405 nm.

An additional, more limited series of experiments was performed with the objective of determining the effects of the higher concentrations (5 and 10 µM) of both dabigatran and rivaroxaban on the reactivity of neutrophil-derived elastase. In this experimental setting, the test agents were added retrospectively to PMNL-free supernatants following activation of the cells with fMLP/CB for 10 min at 37 °C, followed by addition of the elastase substrate and spectrophotometric assessment of enzyme activity.

### 2.5. Spectrofluorimetric Measurement of Cytosolic Ca^2+^

Fura-2-acetoxymethyl ester (fura-2AM) was used as the fluorescent, Ca^2+^-sensitive indicator for these experiments. PMNL (1 × 10^7^/mL) were incubated with fura-2AM (2 μM) for 30 min at 37 °C in PBS, washed and re-suspended in HBSS. The fura-2-loaded cells (2 × 10^6^/mL) were then pre-incubated for 10 min at 37 °C in the presence or absence of dabigatran or rivaroxaban (1–10 μM only) after which they were transferred to disposable reaction cuvettes, which were maintained at 37 °C in a Hitachi 650 10 S fluorescence spectrophotometer with excitation and emission wavelengths set at 340 and 500 nm, respectively. After a stable baseline was obtained, fMLP (1 μM) was added and alterations in fluorescence intensity were monitored over a 5–10 min time course and intracellular Ca^2+^ concentrations (µM) calculated as previously described [18].

### 2.6. Spectrofluorimetric Detection of Neutrophil Extracellular Traps (NETs)

PMNL (1 × 10^6^/mL in a final volume of 4 mL HBSS) were pre-incubated for 10 min at 37 °C in the presence or absence of dabigatran or rivaroxaban (5 and 10 µM only) followed by the addition of PMA (6.25 ng/mL) to activate NETosis. Tubes were then incubated for 90 and 120 min at 37 °C after which the cells were removed by centrifugation. The cell-free supernatants (3 mL) were then mixed with 3 μL of the DNA-binding fluorophore, SYTOX^®^ Orange (5 μM final, Life Technologies, Carlsbad, CA, USA) [19], transferred to cuvettes, and placed in the cuvette holder of a Hitachi 650 10S fluorescence spectrophotometer (Hitachi Ltd., Tokyo, Japan) with the excitation and emission wavelengths set at 530 and 590 nm respectively followed by measurement of fluorescence intensity. The results are expressed as metered fluorescence units (MFUs) following subtraction of the values of time-matched background control systems, which were held at room temperature (25 °C).

### 2.7. Cell Viability

PMNL (1 × 10^6^/mL) were treated with dabigatran or rivaroxaban at a final concentration of 10 μM for 120 min at 37 °C, followed in succession by a 10 min exposure of the cells to propidium iodide (DNA prepstain, Beckman Coulter, Miami, FL, USA, 50 μg/mL) at room temperature and flow cytometric detection of uptake of propidium iodide as a marker of cell membrane damage expressed as % viable cells.

### 2.8. Expression and Statistical Analysis of Results

The results of each series of experiments using PMNL from a minimum of three different donors are expressed as the mean values ± standard errors of the means (SEMs). Statistical significance was determined using the Wilcoxon matched pairs test.

## 3. Results

### 3.1. Generation of Reactive Oxygen Species (ROS) and Release of Elastase

**Figure 1:**
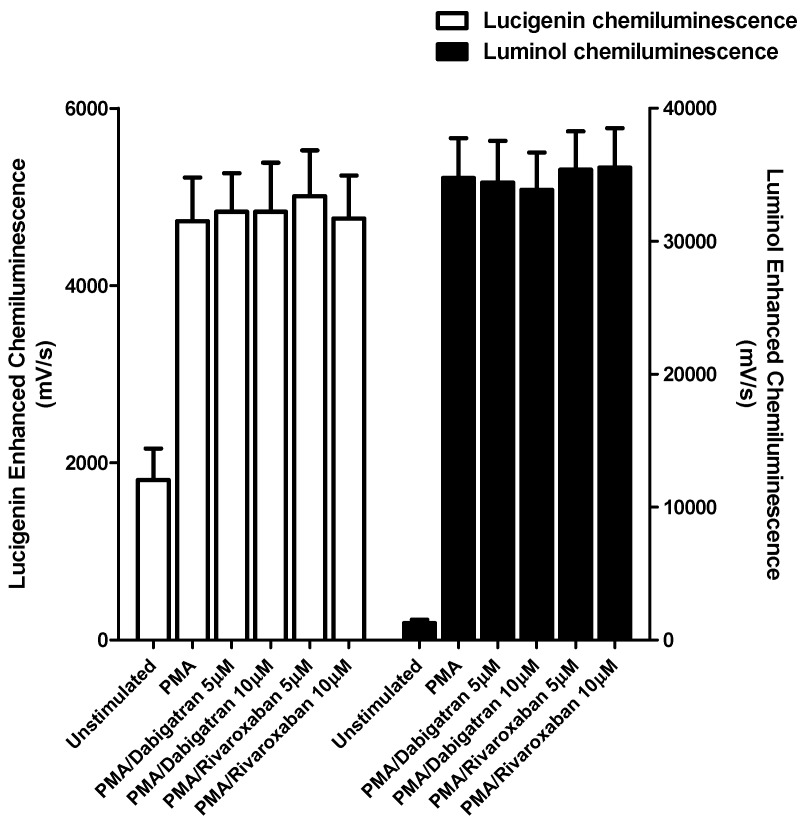
Effects of dabigatran and rivaroxaban (5 and 10 μM) on the PMA-activated lucigenin- and luminol-enhanced chemiluminescence responses of human neutrophils. The results of three different experiments using cells from three different donors are expressed as the mean peak values ± SEMs in mV/s.

**Figure 2:**
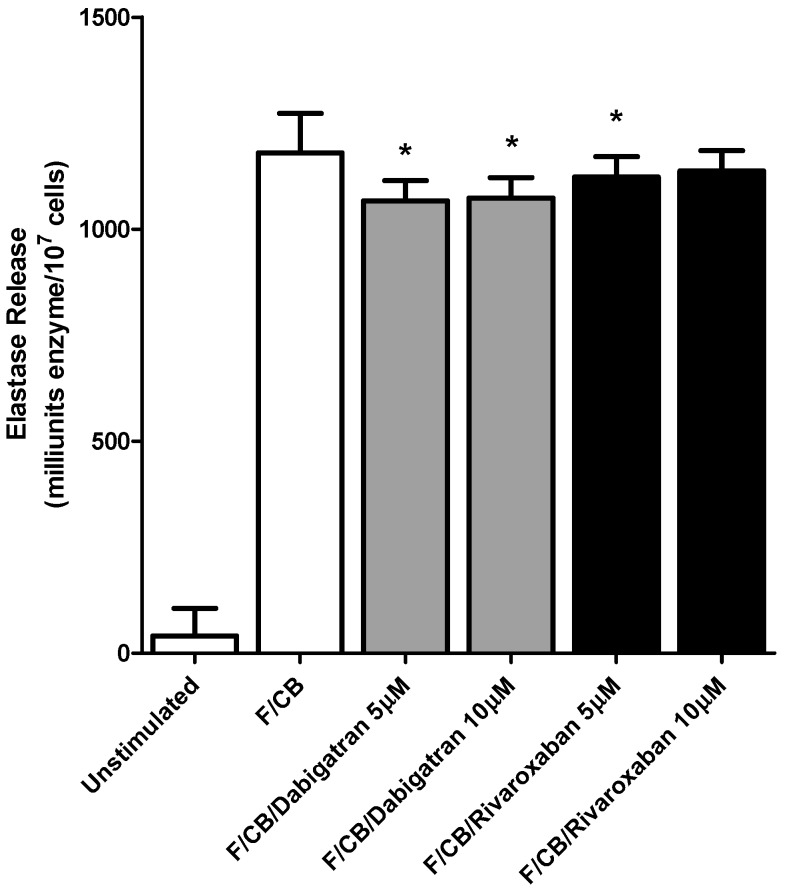
Effects of dabigatran and rivaroxaban (5 and 10 μM) on the release of elastase from *N*-formyl-l-methionyl-l-leucyl-l-phenylalanine/cytochalasin B (fMLP/CB) (1 μM/0.5 µM)-activated human neutrophils. The results of four different experiments using cells from four different donors are expressed as the mean values ± SEMs as milliunits enzyme/10^7^ cells. * *p* < 0.008–*p* < 0.04.

These results are shown in Figure 1 and Figure 2.

Neither of the test agents at the highest concentrations used (5 and 10 μM) affected either the generation of superoxide or myeloperoxidase-derived ROS by PMA-activated PMNL (Figure 1). Similarly, no effects on these PMA-activated responses of PMNL were detected at lower concentrations (0.5 and 1 µM) of either test agent, while basal (unstimulated) responses were also unaffected at all concentrations tested (not shown).

These agents at higher concentrations, dabigatran (5 and 10 µM) and rivaroxaban (5 µM) did however have a modest, albeit statistically significant, inhibitory effect on elastase release from fMLP/CB-activated PMNL; *p* < 0.016 and *p* < 0.008 respectively for dabigatran and *p* < 0.04 for rivaroxaban. (Figure 2). No effects on these fMLP/CB-activated responses of PMNL were evident at lower concentrations (0.5 and 1 µM) of both test agents, while basal responses were also unaffected at all concentrations tested.

To investigate possible direct inhibitory effects of the higher concentrations (5 and 10 µM) of dabigatran and rivaroxaban on the reactivity of elastase, these agents were added retrospectively to supernatants prepared from control neutrophils following activation with fMLP/CB and incubated for 10 min followed by addition of enzyme substrate and monitoring of enzyme activity. No statistically significant effects of either test agent on enzyme activity were detected, the results being 1285 ± 20, 1237 ± 22, 1231 ± 19, 1259 ± 18 and 1289 ± 20 milliunits enzyme activity for the control system and systems treated with dabigatran at 5 and 10 µM and rivaroxaban at 5 and 10 µM respectively (data from two donors with 5 replicates for each).

### 3.2. Cytosolic Ca^2+^ Fluxes

**Figure 3:**
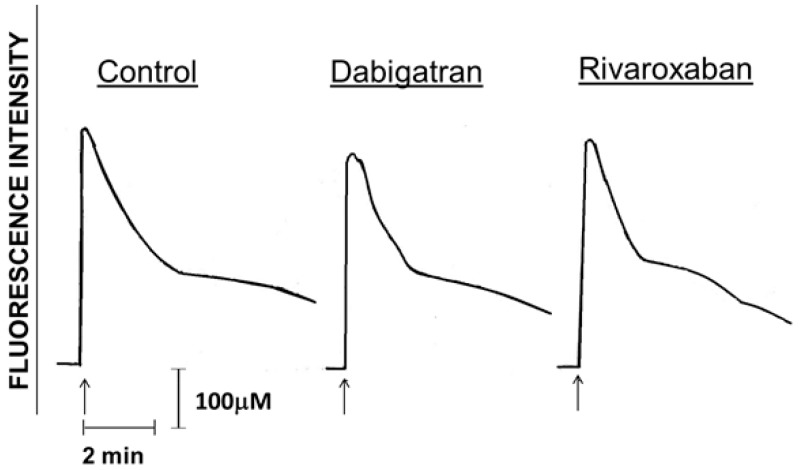
Traces from a typical experiment (3 in the series) using cells from different donors (3 in the series) showing the absence of effects of dabigatran and rivaroxaban (10 µM) on alterations in cytosolic Ca^2+^ concentrations following activation of the cells with fMLP (1 μM) as indicated by the arrow (↑).

These results are shown in Figure 3 and Table 1.

Those in Figure 3 are traces from a single representative experiment, showing that activation of PMNL with fMLP (1 μM) in both the drug-free control and dabigatran-/rivaroxaban (10 µM)-treated systems resulted in the characteristic abrupt increase in fura-2 fluorescence coincident with the release of Ca^2+^ from intracellular storage vesicles. This was followed 10–20 s later by a sharp decline in fluorescence intensity, corresponding with clearance of cytosolic Ca^2+^ and a more gradual decline thereafter corresponding with store-operated uptake of the cation [20]. These events were unaffected by treatment of PMNL with either of the test agents. Data from the complete series of experiments showing basal and peak intracellular Ca^2+^ concentrations measured before and after the addition of fMLP respectively to control and drug-treated (1, 5 and 10 µM) systems are shown in Table 1. No significant effects of dabigatran or rivaroxaban on either basal or peak responses were detected at any of the concentrations tested.

### 3.3. NET Formation

The effects of dabigatran and rivaroxaban (5 and 10 µM) on both spontaneous and PMA-activated NETosis are shown in Table 2.

These results demonstrate lack of effect of both agents on this process.

### 3.4. Neutrophil Viability

The mean viabilities (%) of control PMNL and cells treated with either 10 μM dabigatran or 10 μM rivaroxaban were 98.0 ± 0.2, 97.8 ± 0.3, and 98.0 ± 0.2 respectively (n = 2), demonstrating lack of effects of both test agents on neutrophil viability.

## 4. Discussion

This study examined the effects of the novel oral anticoagulants, dabigatran and rivaroxaban, on various activities of human PMNL in vitro. Neither drug affected the production of ROS, NETosis or cytosolic Ca^2+^ fluxes (which could initiate inflammatory events) following activation of PMNL, nor did they alter basal responses or cell viability. However, degranulation, measured according to the release of the primary granule enzyme, elastase, was modestly, albeit significantly, decreased following treatment of the cells with dabigatran, and, to a lesser extent, rivaroxaban, at the highest concentrations of each agent tested. As neither agent affected enzyme activity in a cell-free system, the inhibitory effects observed when using intact cells appear to result from true inhibition of degranulation by a mechanism which remains to be established.

The aforementioned observations do not, however, exclude the possibility that prolonged exposure to dabigatran and/or rivaroxaban, at incubation times greater than those used in the current study, may alter the functions of PMNL. This seems unlikely, however, given the lack of effects of the higher concentrations of the test agents used in this study. In addition, we concede that this in vitro laboratory-based study may not be entirely representative of the therapeutic setting in which the pleiotropic effects of the test agents, together with those of thrombin, are operative [21,22]. In this context, it is possible that dabigatran, in particular, which inhibits thrombin activity rather than the generation of the active enzyme, may directly activate PMNL by alternative mechanisms to those investigated here. The pleiotropic effects of this agent include augmentation of the interaction of thrombin with protease-activated receptor 1 (PAR-1), resulting in excessive release of PMNL-derived pro-inflammatory cytokines. [21,22]. In addition, dabigatran may also possess pro-thrombotic potential by inducing thrombogenesis via suppression of the thrombin-induced negative-feedback system through inhibition of protein C activation, an effect not seen with the factor Xa inhibitors [14,15].

Interestingly, and somewhat paradoxically, the increase in thrombin generation induced by dabigatran-mediated inhibition of protein C has no effect on fibrinolysis in vitro, even though thrombin-mediated activation of thrombin activatable fibrinolysis inhibitor (TAFI) is unaffected [23]. In the case of endothelial cells, the expression of genes which are strongly up-regulated by thrombin include ELAM-1, VCAM-1, ICAM-1, MCP-1, IL-8, CXCL1, and CXCL2 [24], which, in turn, is likely to promote endothelial adhesion and activation of PMNL and monocytes. In contrast, by blocking thrombin generation, factor Xa antagonists may be anti-inflammatory [24].

Thrombin, via platelet activation, also induces up-regulation of the expression of platelet adhesion molecules, including CD62P, which promotes platelet-driven activation of PMNL [25], as well as CD40, also an important mediator of platelet-leukocyte aggregation, which promotes release of chemokines such as (C-X-C motif) ligand 4 [26]. As such, both of these platelet adhesion molecules play critical roles in the activation and recruitment of leukocytes, as well as in the activation of endothelial cells [25,26]. In addition, dabigatran may also directly increase platelet reactivity by enhancing thrombin receptor density on platelets [27].

Adding to the complexity of the effects of these agents in vivo, the pro-inflammatory effect of thrombin may actually be ameliorated by its interaction with thrombomodulin, even though this pathway may be inhibited in the presence of a damaged endothelium. Activation of protein C is enhanced by an endothelial protein C receptor (EPCR) in large vessels. Activated protein C/EPCR complexes bind to PMNL via the β_2_-integrin, CR3, and PAR-3 and translocate to the nucleus modulating NF-ЌB activity [28].

Numerous studies have been published attempting to prove or refute the contention that the NOACs, especially dabigatran, are associated with an increased incidence of myocardial infarction or atherosclerosis. As mentioned above, however, the pleiotropic effects of these agents, as well as those of thrombin, confound this issue, and, indeed, they may in fact reduce atherogenesis. For example apixaban reduces the inflammatory response in acute stroke, while dabigatran effectively inhibits platelet aggregation in this clinical setting [29]. Similarly, dabigatran may be associated with a reduction in plasma apoB levels in mice, this being an unexpected inhibitory effect on atherogenesis that in the authors’ words was “quite striking” [30].

Clearly, the effects of NOACs are still not fully elucidated. There are pro- and anti-inflammatory effects, as well as pro- and anticoagulant effects, the consequence of which is dependent upon the interplay between various mediators in vivo. In this regard the jury is still out. What can be said is that neither of the test agents investigated in the current study was found to directly potentiate the pro-inflammatory activities of PMNL in vitro, apparently excluding the involvement of these mechanisms in the pathogenesis of cardiovascular events. Finally, however, the real world evidence currently points to NAOCs as being safe and effective anticoagulants [31].

## Figures and Tables

**Table 1:** Effects of treatment of polymorphonuclear leukocytes (PMNL) with dabigatran and rivaroxaban (1–10 µM) on basal and fMLP-activated peak intracellular Ca^2+^ concentrations.

System	Intracellular Ca^2+^ Concentrations (µM)	
	Basal	Peak
Control neutrophils	70.3 ± 4	397.0 ± 36 *
Neutrophils + 1 µM dabigatran	68.3 ± 12	403.9 ± 36
Neutrophils + 5 µM dabigatran	70.3 ± 3	402.6 ± 36
Neutrophils +10 µM dabigatran	64.3 ± 12	392.8 ± 59
Neutrophils + 1 µM rivaroxaban	74.8 ± 13	421.0 ± 34
Neutrophils + 5 µM rivaroxaban	82.0 ± 10	393.4 ± 32
Neutrophils + 10 µM rivaroxaban	68.8 ± 7	415.0 ± 32

* The results of three experiments using cells from three different donors are expressed as the mean values ± SEMs.

**Table 2:** Effects of dabigatran and rivaroxaban on spontaneous and PMA-activated NETosis.

System	NETosis (Metered Fluorescence Units)	
	Spontaneous	PMA-Activated
Control neutrophils	29.5 ± 4.1 *	231.8 ± 13.6
Neutrophils + 5 μM dabigatran	19.5 ± 2.5	238.3 ± 15.6
Neutrophils + 10 μM dabigatran	26.8 ± 5.2	254.0 ± 28.5
Neutrophils + 5 μM rivaroxaban	21.0 ± 5.0	250.3 ± 39.7
Neutrophils + 10 μM rivaroxaban	34.5 ± 7.6	260.0 ± 15.7

* The results of three experiments using cells from three different donors are expressed as the mean values ± SEMs.
